# E-Cigarette Battery Explosions: Review of the Acute Management of the Burns and the Impact on Our Population

**DOI:** 10.7759/cureus.5355

**Published:** 2019-08-09

**Authors:** Luis Quiroga, Mohammed Asif, Tomer Lagziel, Deepa Bhat, Julie Caffrey

**Affiliations:** 1 Surgery, Burn Center, The Johns Hopkins University School of Medicine, Baltimore, USA; 2 Surgery, The Johns Hopkins University School of Medicine, Baltimore, USA; 3 Medicine, Burn Center, The Johns Hopkins University School of Medicine, Baltimore, USA

**Keywords:** electronic nicotine delivery devices, explosions, burns, electronic cigarette, lithium batteries, thermal runaway, combustion

## Abstract

Electronic cigarettes, also known as e-cigarettes (E-cig), are lithium-battery-powered devices, which became available for sale in the United States in 2017. It has gained significant popularity among younger-generation tobacco smokers due to its advertisement as a non-toxic inhalation property and a potential smoking-cessation aid. The US Food and Drug Administration (FDA) has been regulating e-cigarettes as tobacco products and not as drug-delivery devices, as many medical experts think it should be categorized. In the last few years, the medical community has encountered increasing episodes of burn injuries secondary to e-cigarette battery explosion. Explosions occur through a process known as a "thermal runaway.” This process occurs when the battery overheats and the internal battery temperature increases dangerously high, to the point of inner fire and explosion. Overcharge, puncture, external heat, short circuit, amongst others, are conditions that cause a “thermal runaway.”

This is a retrospective review and analysis of six patients with superficial, partial, and full-thickness burn injuries related to e-cigarette battery explosions managed at Johns Hopkins Bayview Burn Center over the course of one year. Lund-Browder diagrams and calculations were used to assess the total body surface area (TBSA) burns. Laser Doppler imaging (LDI) was used to evaluate the indeterminate depth of the burn.

Only one of our six patients required tangential excision and skin grafting. The rest of our patients were treated conservatively with complex wound care, which included the mixed combination of topical collagenase and bacitracin, collagenase and mafenide, or silver sulfadiazine as a single-agent treatment with an excellent response. Five patients were discharged home within a week, including the patient who required operative excision and auto-grafting. One patient stayed for eight days for pain control and complex wound care.

Our experience with these burns has been similar to what is previously reported. Most of these burns are managed with complex wound care without any surgical interventions. The e-cigarette batteries seem more prone to failure due to an inherent weakness in their structural design. This makes them particularly susceptible to the “thermal runaway.” Therefore, we recognized the need to expand the regulation and control of the quality of these devices. Prevention of these burns will require continuing education for the community on the use of E-cig. products and its potential hazardous implications.

New efforts should be made to educate the community and healthcare providers regarding the potential hazardous implication of carrying these batteries. Also, there is insufficient data to support or deny the long-term health effects of using e-cigarettes.

## Introduction

Electronic cigarettes, also known as e-cigarettes, are lithium-battery-powered devices that produce a heated aerosol (or vapor) for recreational smoking. Unlike traditional cigarettes, there is no burning of tobacco leaves that occurs or tar produced. Typically, smokers use nicotine, cannabis, hash, or other simple flavor vapors. It is commonly referred to as “vaping,” in contrast to smoking, because combustion does not occur. Commercialization has produced many different models with various shapes that differ on how much nicotine can be released with each inhalation.

Many resemble traditional cigarettes, cigars, and pipes, and prices for e-cigarettes range from $30 to $300. In 2007, e-cigarettes became available for sale on the United States market and since then, their use has grown among smokers [1]. One of the main reasons for its current popularity is the perception of consumers that vaping is healthier than smoking traditional cigarettes [1-2]. Since initial commercialization, companies have advertised the absence of inhaled carcinogenic substances and combustion when compared to regular cigarettes. Some companies have even promoted e-cigarettes as a smoking cessation aid.

However, there is insufficient data to deny or support long-term health effects [1-2]. Additionally, in many countries, government regulators are unsure how to classify these relatively new devices. For example, the U.S. Food and Drug Administration (FDA) has been regulating e-cigarettes as tobacco products and not as drug-delivery devices, as many medical experts think it should be categorized [3].

In the last few years, the medical community has encountered increasing episodes of burn injuries secondary to e-cigarette battery explosions [4-5]. Although battery failure and explosion have been well-documented in different lithium batteries, including cellphones and laptop computers, e-cigarette batteries seem more prone to failure due to an inherent weakness in their structural design. The cylindrical shape of many of these batteries creates a weak point on the ends where the battery’s seal is placed after filling it with electrolyte. Lithium batteries are typically composed of four elements: a negative electrode or an anode (made of copper and carbon material); a positive electrode or a cathode (made of aluminum and lithium-ion); an electrolyte, such as a lithium salt (LiPF, LiBF, or LiCIO); an organic solvent (ethylene carbonate, dimethyl carbonate, or diethyl carbonate), and a porous plastic membrane separator.

Thermal runaway, or the overheating of the battery, is the process by which the internal battery temperature increases to the point where an internal fire or explosion can be started by conditions such as overcharge, puncture, external heat, a short circuit, etc.

## Case presentation

Sequence of a typical failure

**Figure 1:**
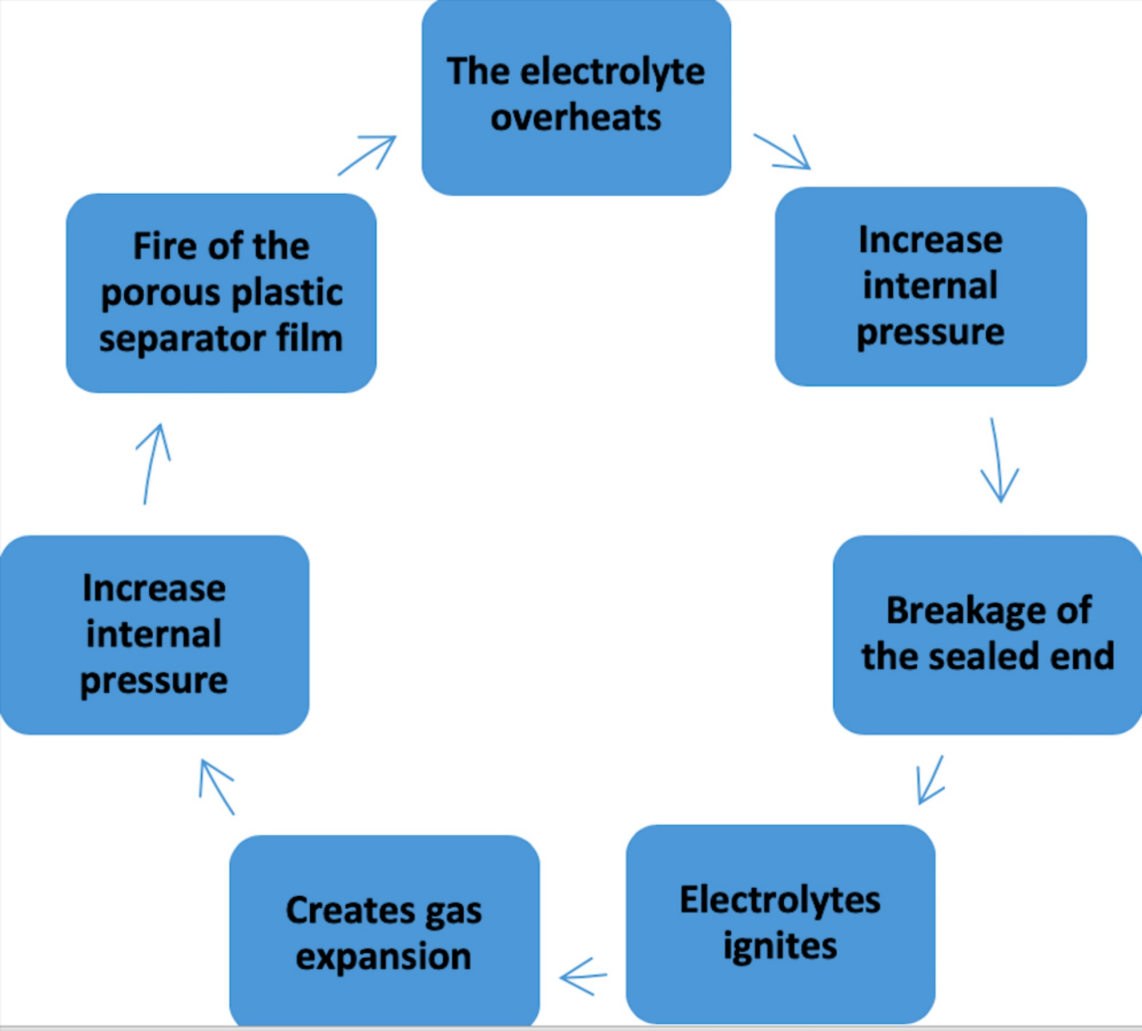
Sequence of a typical failure: “thermal runaway.”

The electrolyte overheats -> Increased internal pressure -> Breakage of the sealed end -> Electrolyte ignites -> Creates gas expansion -> Increased internal pressure -> Combustion of the porous plastic separator film (Figure 1).

**Figure 2:**
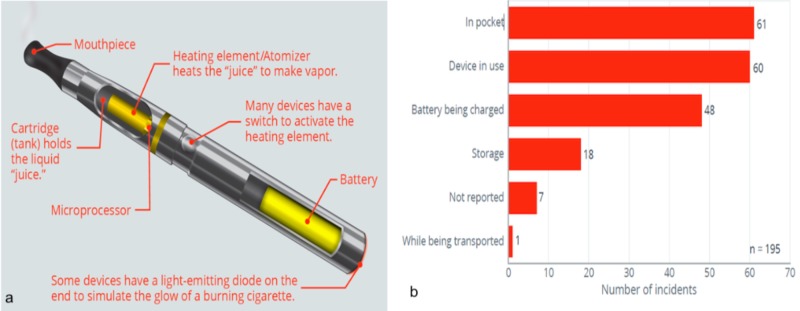
Parts of an electronic cigarette (a) and statuses of e-cigarettes when the incident occurred (b).

While most of these failures and explosions have occurred while charging the lithium battery, several have occurred when a person has been carrying the device and/or battery in a pocket or even when using the device [5] (Figure 2b). Most of these burns are caused by a combined mechanism of flame and chemical burn. The chemical burn is thought to be caused by lithium salt, which is an alkaline substance.

The following are reports of our experience dealing with these kinds of injuries. In the last year, we have managed a total of six cases that makes our current report the largest series of cases in the literature.

Case 1

A 27-year-old male was admitted to our burn center after sustaining a 6% indeterminate partial-thickness burn (2nd degree) to bilateral hands and the lateral aspect of his right thigh. The patient reported that his right pant pocket, containing only the e-cigarette battery, spontaneously caught fire. He also sustained superficial partial-thickness burns to the dorsum of his right hand while trying to put the fire out.

He underwent debridement at the bedside to remove dead tissue and any residue in order to reduce the risk of infection and any inflammatory response. He was treated with local wound care using Xeroform and Bacitracin dressings. His wound was greater than 85% re-epithelialized two weeks post-injury.

Case 2

**Figure 3:**
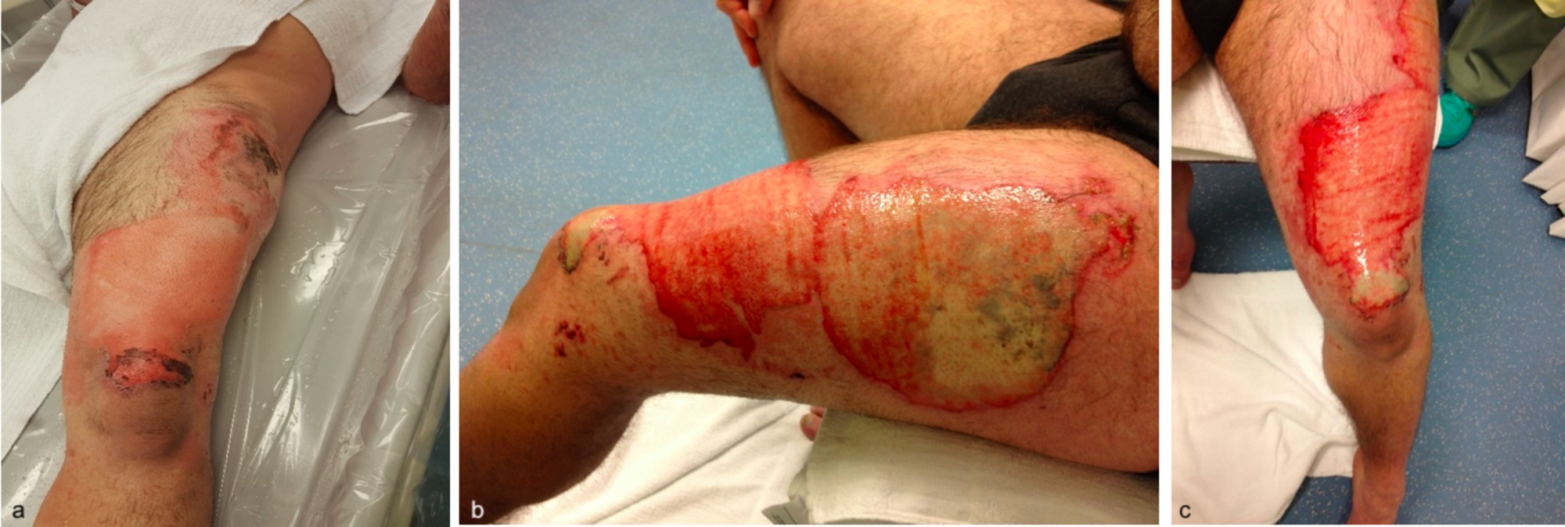
Left thigh burns: initial evaluation (a), and post 48 hours (b, c).

A 36-year-old male presented to our burn center after sustaining a 3% indeterminate partial-thickness burn (2nd degree) to his left hand and anterolateral aspect of his left thigh and knee (Figure 3). He reported that he had two batteries for an e-cigarette device in his left pocket when he suddenly felt heat and noticed that his pants were on fire. He sustained superficial partial-thickness burns to his left palm while trying to put out the fire.

**Figure 4:**
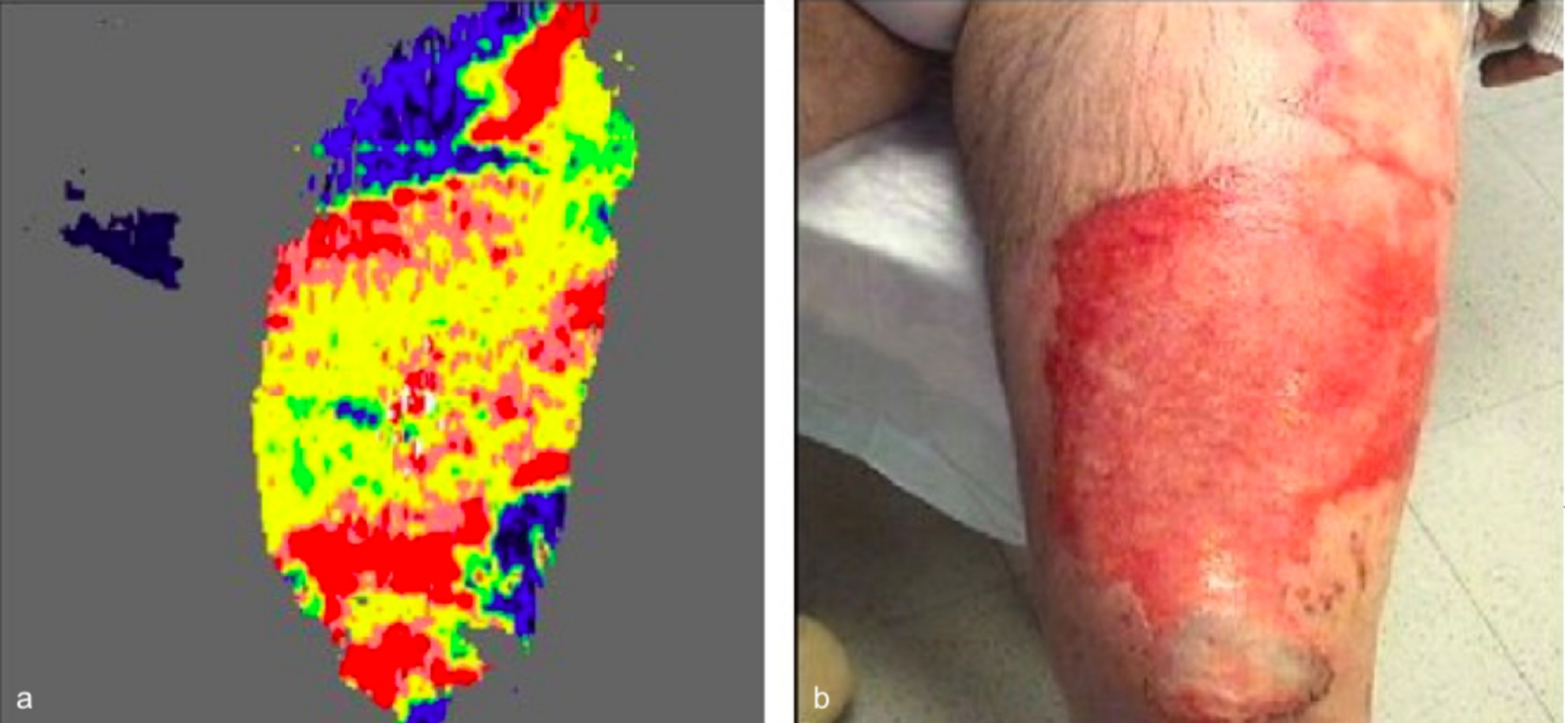
Laser Doppler imaging (LDI) performed 48 hours post-injury on anterior aspect of the left thigh and knee. LDI (a) and photo (b).

**Figure 5:**
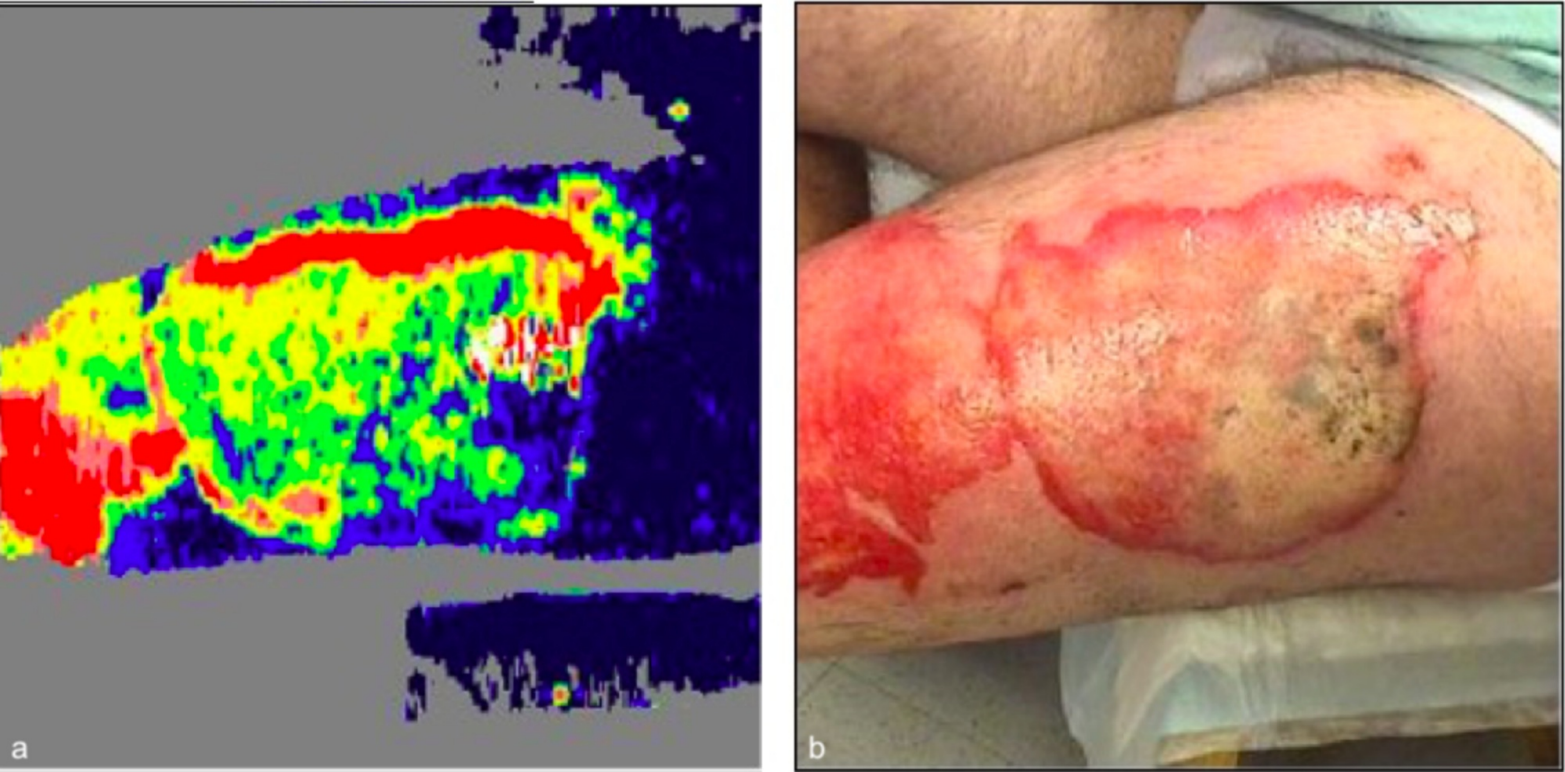
Laser Doppler Imaging (LDI) performed 48 hours post-injury on the lateral aspect of the left thigh. LDI (a) and photo (b).

Laser Doppler imaging (LDI) was performed 48 hours post-injury. His left anterior thigh wound scans revealed a coloring map consistent with adequate perfusion of the tissue, suggesting a good healing potential of 14 days on the edges and 14-21 days for the remainder of the wound (Figures 4-5).

He opted to forgo surgical excision and undergo conservative management of his complex wound burn. He was treated with a combination of Sulfamylon (mafenide acetate) and Santyl (collagenase) three times per day for 5 days. He was discharged home and 3 weeks post-injury follow up visit demonstrated completely healed thigh wound. 

Case 3

A 27-year-old man presented with 6% TBSA superficial partial burn to left thigh after an e-cigarette erupted into flames. The patient reported that he was at work when he heard a loud "hissing" sound that startled him, then he felt a burning pain in his lower extremity followed by noticing his pants were on fire. The patient removed his clothing which he reported had melted to his skin. He was carrying a lithium battery for his e-cigarette in his pants pocket. He sustained superficial burns to his bilateral hands while trying to extinguish the flames. 

**Figure 6:**
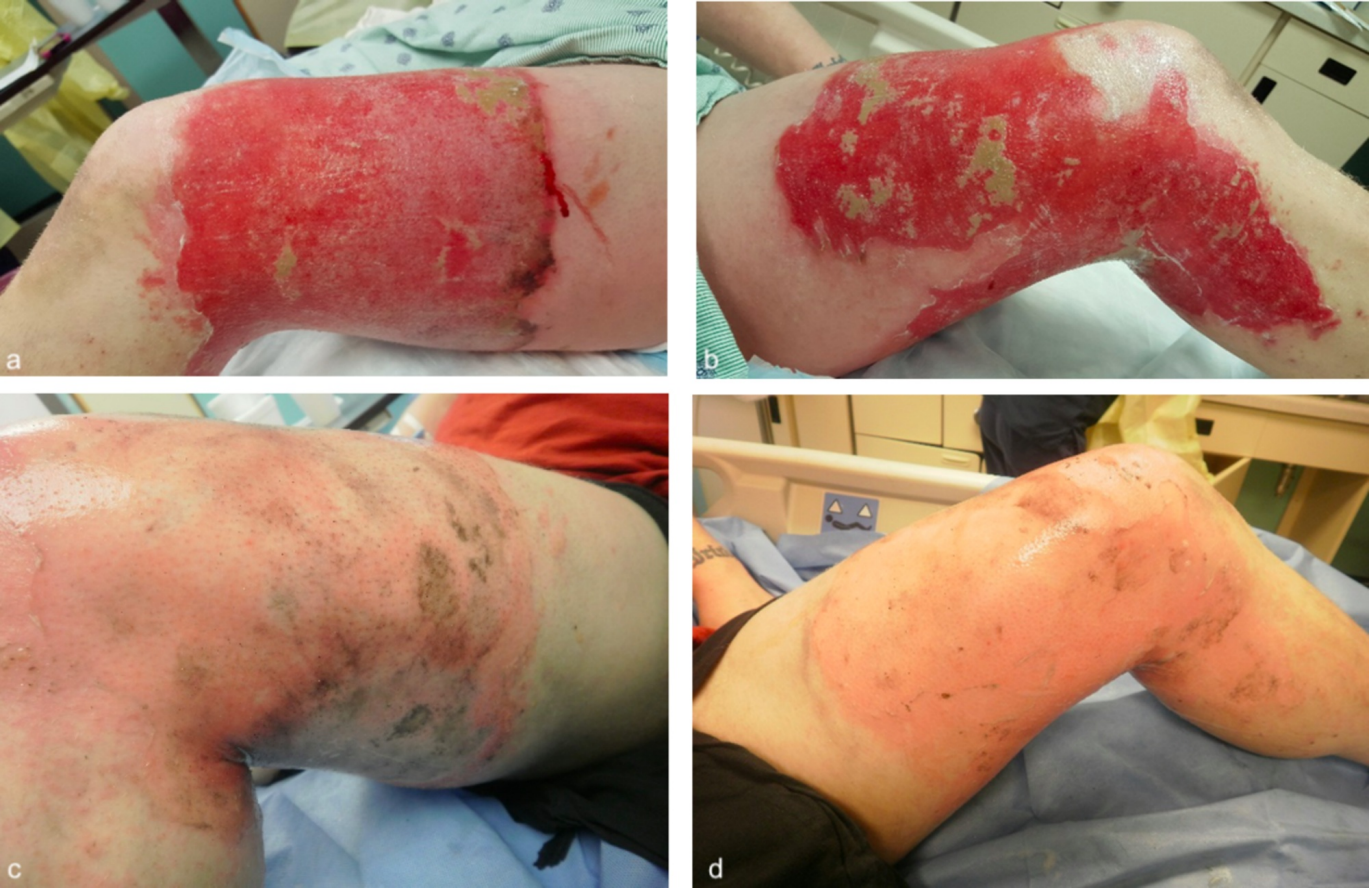
Injuries before treatment (a-b) and after two weeks of treatment (c-d).

He was treated with silver sulfadiazine (Silvadene) to his left thigh and his wounds healed within 2 weeks (Figure 6). 

Case 4

**Figure 7:**
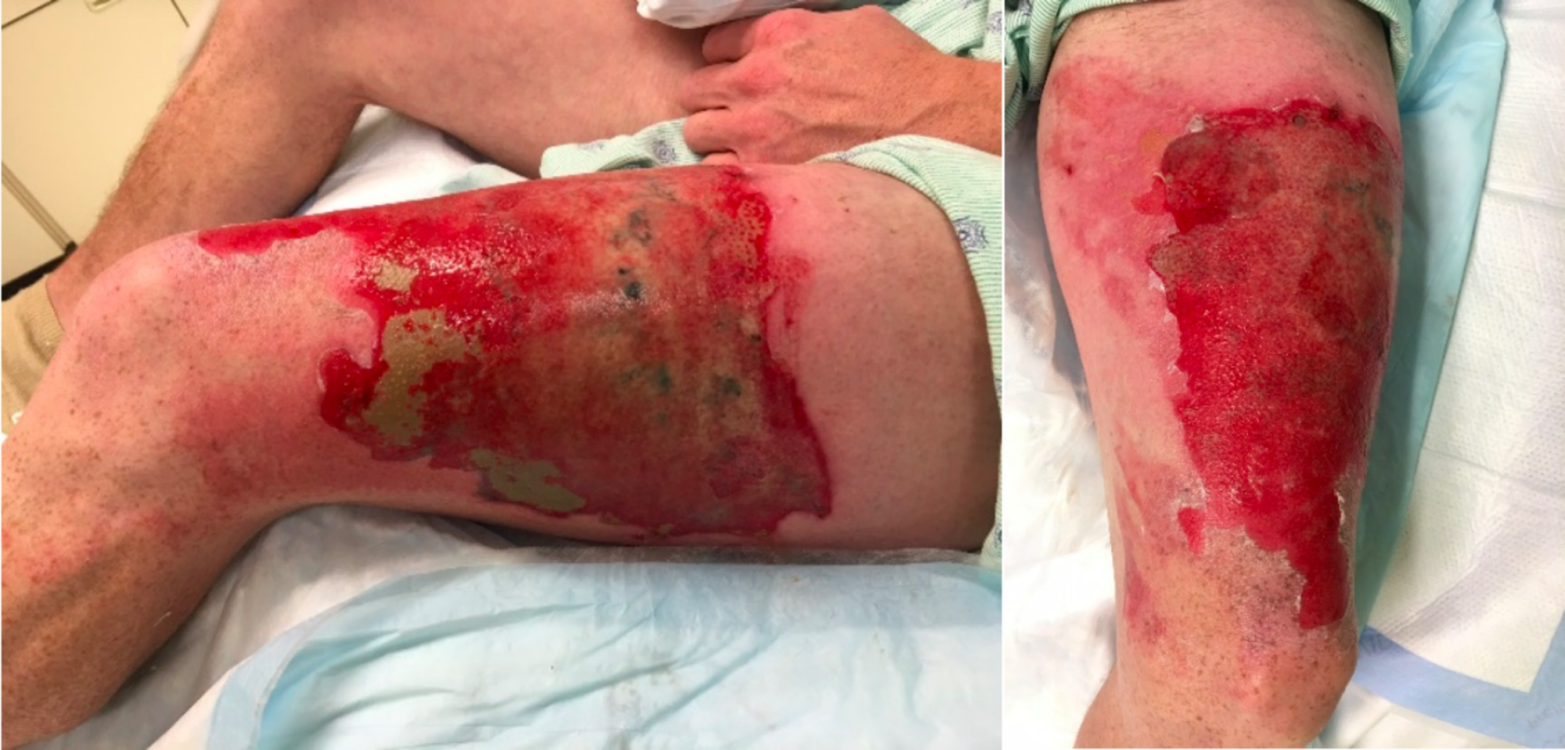
Burn injuries upon admission.

A 36-year-old man was admitted with 2% TBSA partial superficial thickness burn to his left thigh (Figure 7). The patient was carrying an e-cigarette's battery that exploded in his pocket and caused him burns. He was treated with mechanical debridement followed by Xeroform and Bacitracin dressing changes daily. His wounds were completed healed 2 weeks post-injury.

Case 5

A 46-year-old man presented with 3% TBSA deep and superficial partial thickness burn to his right thigh from a battery explosion. He reported that he was carrying the battery for his electronic cigarette in his right shorts pocket when it spontaneously exploded causing flame burns to his right thigh. He underwent tangential excision and split-thickness skin grafting to the right thigh on hospital day 4. Dressings were taken down on postoperative day 4 and 100% graft take was noted. The patient was followed up in clinic and completely healed incorporated graft was noted 5 weeks post-injury.

Case 6

**Figure 8:**
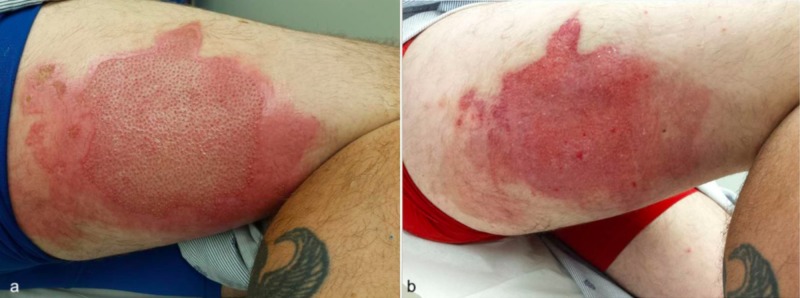
Burn injuries upon admission (a) and after four days of Bacitracin and Santyl treatment (b).

A 32-year-old male presented to the emergency room with 4% indeterminate burn of the right posterior thigh. His injury occurred when his e-Cigarette in his pocket accidentally ignited resulting in contact/thermal injury. He was treated with Bacitracin and Santyl combination twice daily for 4 days (Figure 8). An LDI was performed and demonstrated good healing potential except for a small area with deep tissue injury within the large wound. His wounds are completely healed 6 weeks post-injury.

## Discussion

E-cigarettes have been increasing in popularity in recent years, likely due to the fact that consumers perceive vaping to be healthier than smoking. Further studies are required to support the long-term health benefits. Additionally, many regulators are unsure how to classify these relatively new devices and what the standards should be in the manufacture as well as quality control.

Even though lithium battery failures and instability are rare events, they have been well-documented in different devices. The particular build-shape of the e-cigarette battery seems to make them particular susceptible to this kind of failure and thermal runaway [6].

In the last few years, the medical community has encountered increasing episodes of burn injuries secondary to e-cigarette battery explosion that has been reflected by the media and news. However, there are no clear published guidelines for managing lithium battery burns. In our series, we were able to manage the majority of our patient’s injuries at the bedside, with varying combinations of mechanical/chemical debridement as well as topical wound care. Only one of our six patients required tangential excision and skin grafting. The rest of our patients were treated conservatively with complex wound care with excellent responses.

Previous case reports in the literature have pointed out that these burns are a combination of a flame burn with some component of chemical burn due to residual elemental lithium reacting with water by producing alkali lithium hydroxide and hydrogen gas [7]. However, our patients did not show exacerbation of their symptoms after debridement and irrigation with sterile water.

Although not always necessary, Laser Doppler imaging can be a useful adjuvant tool for indeterminate burn wounds. The clinical exam is, unfortunately, only accurate in about two-thirds of cases when determining burn depth in partial-thickness burns [8]. It has been well-established that LDI predicts healing time for wounds with a very high degree of accuracy (95%-100%) [9]. In our patient’s case, LDI aided us in determining whether or not his wounds would heal or would necessitate surgical debridement of the wound. Since the results of LDI suggested that the wound had good healing potential, we felt that the patient’s wishes to forego any surgical debridement were acceptable and would not hinder his wound healing/recovery.

## Conclusions

As e-cigarette use is climbing in the general population, clinicians need to attune themselves to the presentation and management of these injuries. As presented in this case series, there is a spectrum of treatment choices and therapy should be tailored to each individual patient. The majority of our patients were managed with local wound care and without the need for procedural intervention. LDI is a useful tool in guiding clinical judgment when it is difficult to assess the burn wound depth based solely off of clinical exam.
